# Clinical and molecular characterization of patients affected by Beckwith‐Wiedemann spectrum conceived through assisted reproduction techniques

**DOI:** 10.1111/cge.14193

**Published:** 2022-07-21

**Authors:** Diana Carli, Matteo Operti, Silvia Russo, Guido Cocchi, Donatella Milani, Chiara Leoni, Elisabetta Prada, Daniela Melis, Mariateresa Falco, Jennifer Spina, Vera Uliana, Osimani Sara, Fabio Sirchia, Luigi Tarani, Marina Macchiaiolo, Flavia Cerrato, Angela Sparago, Laura Pignata, Pierpaola Tannorella, Simona Cardaropoli, Andrea Bartuli, Andrea Riccio, Giovanni Battista Ferrero, Alessandro Mussa

**Affiliations:** ^1^ Department of Public Health and Pediatric Sciences University of Torino Torino Italy; ^2^ Research Laboratory of Medical Cytogenetics and Molecular Genetics IRCCS Istituto Auxologico Italiano Milan Italy; ^3^ Neonatology Unit, St. Orsola‐Malpighi Polyclinic, Department of Medical and Surgical Sciences (DIMEC) University of Bologna Bologna Italy; ^4^ Fondazione IRCCS Ca' Granda Ospedale Maggiore Policlinico Milan Italy; ^5^ Center for Rare Diseases and Birth Defects, Department of Woman and Child Health and Public Health Fondazione Policlinico Universitario A. Gemelli IRCCS Rome Italy; ^6^ Department of Medicine, Surgery and Dentistry “Scuola Medica Salernitana” University of Salerno Fisciano Italy; ^7^ Pediatric Unit San Giovanni di Dio e Ruggi D'Aragona University Hospital Salerno Italy; ^8^ Medical Genetics Unit University Hospital of Parma Parma Italy; ^9^ Department of Pediatrics Scientific Institute San Raffaele Milan Italy; ^10^ Unit of Medical Genetics, Department of Diagnostic Medicine Fondazione IRCCS Policlinico San Matteo Pavia Italy; ^11^ Department of Molecular Medicine University of Pavia Pavia Italy; ^12^ Department of Pediatrics, Medical Faculty “Sapienza” University of Rome Rome Italy; ^13^ Rare Diseases and Medical Genetics, Department of Pediatric Medicine Bambino Gesù Children's Hospital, IRCCS Rome Italy; ^14^ Department of Environmental Biological and Pharmaceutical Sciences and Technologies (DiSTABiF) Università degli Studi della Campania “Luigi Vanvitelli” Caserta Italy; ^15^ Institute of Genetics and Biophysics A. Buzzati‐Traverso Consiglio Nazionale delle Ricerche Naples Italy; ^16^ Department of Clinical and Biological Sciences University of Torino Torino Italy; ^17^ Pediatric Clinical Genetics Unit, Regina Margherita Childrens Hospital Città della Salute e della Scienza di Torino Torino Italy

**Keywords:** assisted reproductive technologies, Beckwith‐Wiedemann spectrum, hypomethylation, imprinting disorders, uniparental disomy

## Abstract

The prevalence of Beckwith–Wiedemann spectrum (BWSp) is tenfold increased in children conceived through assisted reproductive techniques (ART). More than 90% of ART‐BWSp patients reported so far display imprinting center 2 loss‐of‐methylations (IC2‐LoM), versus 50% of naturally conceived BWSp patients. We describe a cohort of 74 ART‐BWSp patients comparing their features with a cohort of naturally conceived BWSp patients, with the ART‐BWSp patients previously described in literature, and with the general population of children born from ART. We found that the distribution of UPD(11)pat was not significantly different in ART and naturally conceived patients. We observed 68.9% of IC2‐LoM and 16.2% of mosaic UPD(11)pat in our ART cohort, that strongly differ from the figure reported in other cohorts so far. Since UPD(11)pat likely results from post‐fertilization recombination events, our findings allows to hypothesize that more complex molecular mechanisms, besides methylation disturbances, may underlie BWSp increased risk in ART pregnancies. Moreover, comparing the clinical features of ART and non‐ART BWSp patients, we found that ART‐BWSp patients might have a milder phenotype. Finally, our data show a progressive increase in the prevalence of BWSp over time, paralleling that of ART usage in the last decades.

## Graphical abstract

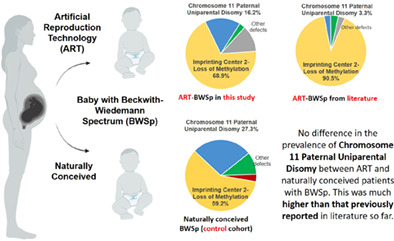

## 1 INTRODUCTION

Birth from assisted reproductive technology (ART) account for approximately 3.1% of all births in Europe^1^ and are known to be associated with pregnancy complications, preterm delivery and related problems,^2^ increased birth defects rate,^3^ long‐term effects on health,^4^ and genetic/epigenetic risk.^5^, ^6^ Most of such adverse events are indeed connected with characteristics of the couples that undergo ART, including age, health condition, and subfertility.^7^ Several studies documented an increased risk of DNA methylation anomalies in children born from ART,^8^, ^9^ some resulting in an higher incidence of human imprinting disorders.^10^ It is unclear whether such epigenetic anomalies are the direct result of ART itself or, rather, connected with genetic/environmental factors causing parental subfertility.^7^, ^11^

The most common human imprinting disorder is Beckwith‐Wiedemann spectrum (BWSp), a congenital overgrowth condition with cancer predisposition and a prevalence of 1:10340 in naturally conceived births,^12^ and 1:1126 in the population born from ART.^6^ BWSp is characterized by a variable association of neonatal macrosomia, postnatal overgrowth, hyperinsulinemic hypoglycemia, abdominal wall defects, macroglossia, lateralized overgrowth, organomegaly, auricular abnormalities, nevus flammeus at the glabella, nephrourological abnormalities and predisposition to the development of embryonal tumors.^13^, ^14^, ^15^, ^16^, ^17^, ^18^ Over 80%^19^ of patients affected by BWSp harbor an epigenetic defect of the imprinted chromosomal region 11p15.5, including hypomethylation of Imprinting Center 2 (IC2‐LoM, nearly 50% of cases), chromosome 11 paternal uniparental disomy (UPD[11]pat, 20% of cases), gain‐of‐methylation of the imprinting center 1 (IC1‐GoM, 10% of cases).^19^ More rare are the genetic defects leading to BWSp, such as loss‐of‐function variants of *CDKN1C* or chromosomal rearrangements of the 11p15.5 region. Each genotype is characterized by a specific phenotype and tumor risk.^17^

The proportion of children with BWSp that are conceived through ART is well above that of the general population, ranging from 4%^20^, ^21^, ^22^ in the earliest reports dating back two decades ago, to 15% in the most recent ones.^23^ Patients with BWSp conceived through ART have been reported to display typically IC2‐LoM (>90% of cases),^6^ suggesting that a defect in imprint establishment or maintenance is underlying the association between BWSp and ART.

To further investigate into this issue, here we describe the genotypic and phenotypic features of a large Italian cohort of patients with BWSp conceived through ART and compare them with (a) a cohort of naturally conceived patients with BWSp, (b) previously reported ART‐BWSp cohorts, and (c) the general population of children born after ART.

## 2 METHODS

This is a retrospective observational study that was conducted on a sample of patients affected by BWSp born after ART diagnosed and followed in 15 pediatric clinical genetic centers in Italy and with the help of the Italian Association of patients affected by BWSp (AIBWS, www.aibws.org). Written informed consent for the study was obtained from patients or guardians for the study, according to the local ethic committee's policy. The study was approved by the ethics committee of the Città della Salute e della Scienza University Hospital of Torino, Italy (IRB approval protocol 0052021–0052712 with ID 155/2022, May2022).

Two criteria were considered for patients' inclusion: diagnosis of BWSp (i.e., with positive molecular tests and/or with clinical diagnosis made with the specific score of the 2018 International Consensus,^19^ with ≥4 points), and conception through ART, including ovarian stimulation, intrauterine insemination (IUI), in vitro fertilization (IVF), or intracytoplasmatic sperm injection (ICSI). Clinical and molecular data were obtained directly from the clinical center where the patients were diagnosed or followed‐up.

Methylation analysis of the chromosomal region 11p15.5 was performed by CoBRA (Combined Bisulfite Restriction Analysis), or MS‐MLPA (Methylation‐Sensitive Multiple Ligation‐dependent Probe Amplification, MRC Holland, Amsterdam, Netherlands^24^). In patients with IC2‐LoM and IC1‐GoM, UPD(11)pat was confirmed by either high‐resolution polymorphism or microsatellite analysis.^25^ Patients scoring negative for 11p15.5 methylation defects underwent *CDKN1C* Sanger sequencing.^26^

For comparison, three cohorts were used: a literature‐derived cohort of ART‐BWSp patients, a naturally conceived (non‐ART)‐BWSp cohort, and a non‐BWSp ART cohort. The literature derived ART‐BWSp cohort was obtained merging previously described ART‐BWSp cohorts: the literature search on Pubmed was conducted to identify publications reporting case series of patients with BWSp conceived through ART (original search string “Beckwith‐Wiedemann Syndrome” [Mesh] and “Reproductive Techniques, Assisted” [Mesh], then refined adding references from retrieved papers^10^, ^20^, ^21^, ^22^, ^23^, ^27^, ^28^, ^29^, ^30^, ^31^, ^32^, ^33^, ^34^, ^35^). The naturally conceived BWSp cohort was our historical one (*n* = 318) was derived from the one previously described by our group,^16^ after exclusion of the conceived through ART (*n* = 14). For comparison between our naturally conceived BWSp cohort and ART‐BWSp cohort, we did not used the data of patients scoring negative to the molecular tests in the current ART‐BWSp cohort, as our historical non‐ART‐BWSp cohort included only molecularly confirmed patients and was published before the definition of BWSp diagnostic criteria.^19^ To compare our study group with the cohort of children born in Italy after ART (ART‐nonBWSp), we used the data reported in the National Registry of Medically Assisted Procreation (www.iss.it/rART, accessed September 15, 2021, covering the years 2005–2019).

Data were compared using the χ^2^ test for distribution analysis of variables greater than 200, the χ^2^ test with Yates's correction for variables between 40 and 200, and Fisher's exact test for variables <40. Comparison between continuous variables was performed with Student's *t* test for variables with normal distribution or Mann–Whitney's U test for those distributed non‐normally, after checking for homoscedasticity of the sample with Shapiro–Wilk test. Correlation between continuous variables was confirmed with Pearson's method. The *p*‐values less than 0.05 were considered statistically significant.

## 3 RESULTS

Our ART‐BWSp cohort included 74 patients, 40 females (54.1%) and 34 males (45.9%), all molecularly tested on blood‐extracted DNA: among them, 65 (87.8%) had a molecular anomaly consistent with BWSp and 9 (12.2%) were negative with a clinical score ≥4.^19^ In Table 1 we report their clinical characteristics, and in Table 2 family history, type of ART, and pregnancy, sorted by molecular subtype. All these data were compared between the IC2‐LoM and UPD(11)pat subgroups; patients with IC1‐GoM or negative molecular test were not included in the comparison because of the low number of cases. We observed that the subgroup with mosaic UPD(11)pat showed an higher frequency of lateralized overgrowth (91.7% vs. 50.1%, *p* = 0.001), malignancies (16.7% vs. 0%, *p* = 0.003), and renal anomalies (41.7% vs. 9.8%, *p* = 0.007). Also, more ART attempts before obtaining a pregnancy were made in the UPD(11)pat than in the IC2‐LoM subgroup (2.6 ± 2.1 vs. 1.1 ± 1.4, *p* = 0.036).

**TABLE 1:** Clinical characteristics of the ART‐BWSp patients' group

	IC2‐LoM	IC1‐GoM	UPD(11)pat	Negative	Total	*p*‐value^a^
*n*	51 (68.9%)	2 (2.7%)	12 (16.2%)	9 (12.2%)	74	–
Females	28 (54.9%)	2 (100%)	6 (50%)	4 (44.4%)	40 (54.1%)	0.759
Males	23 (45.1%)	0 (0%)	6 (50%)	5 (55.6%)	34 (45.9%)	
BWSp score^19^	5.5 ± 2.1	6.5 ± 0.7	6.2 ± 2.4	5.1 ± 1.6	5.6 ± 2.1	0.356
Neonatal hypoglycemia	19 (37.3%)	1 (50%)	5 (41.7%)	6 (66.7%)	31 (41.9%)	0.777
Neonatal hyperinsulinism	1 (2.0%)	1 (50%)	1 (8.3%)	0 (0.0%)	3 (4.1%)	0.257
Macroglossia	42 (82.4%)	1 (50%)	7 (58.3%)	6 (66.7%)	56 (75.7%)	0.072
Abdominal wall defects	34 (66.7%)	1 (50%)	7 (58.3%)	6 (66.7%)	49 (66.2%)	0.586
Omphalocele	7 (13.7%)	0 (0.0%)	2 (16.7%)	0 (0.0%)	9 (12.5%)	0.793
Umbilical hernia or diastasis recti	27 (52.9%)	1 (50%)	5 (41.7%)	9 (100%)	40 (54.1%)	0.482
Lateralized overgrowth	26 (50.1%)	2 (100%)	11 (91.7%)	3 (33.3%)	42 (56.8%)	**0.001** ^b^
Organ enlargement	9 (17.6%)	1 (50%)	3 (25%)	1 (11.1%)	14 (18.9%)	0.559
Ear pits or creases	16 (31.3%)	1 (50%)	7 (58.3%)	6 (66.7%)	30 (40.5%)	0.081
Angioma at the glabella	27 (52.9%)	1 (50%)	4 (33.3%)	5 (55.6%)	37 (50%)	0.222
Polyhydramnios	8 (15.7%)	0 (0.0%)	2 (16.7%)	0 (0.0%)	10 (13.5%)	0.993
Neonatal macrosomia	22 (43.1%)	0 (0.0%)	4 (33.3%)	2 (22.2%)	28 (37.8%)	0.535
Postnatal overgrowth	20 (39.2%)	1 (50.0%)	4 (33.3%)	0 (0.0%)	25 (33.8%)	0.650
Malignant tumors	0 (0.0%)	0 (0.0%)	2 (16.7%)	0 (0.0%)	2 (2.7%)	**0.003** ^b^
Renal anomalies	5 (9.8%)	1 (50.0%)	5 (41.7%)	1(11.1%)	12 (16.2%)	**0.007** ^b^

^a^ The reported *p*‐value represents the result of a comparison between the subgroup with IC2‐LoM and UPD(11)pat.

^b^ Statistically significant.

**TABLE 2:** Family history, kind of technology used, and pregnancy characteristics of the patients with Beckwith‐Wiedemann spectrum conceived after assisted reproduction technology (ART‐BWSp)

		IC2‐LoM (*n* = 51)	IC1‐GoM (*n* = 2)	UPD(11)pat (*n* = 12)	Negative (*n* = 9)	Total (*n* = 74)	*p* ^a^
Presence of siblings		18 (35.3%)	1 (50%)	6 (50%)	3 (75%)	28 (37.8%)	0.345
Time of pregnancy attempts (years)		4.5 ± 3.5	1.5	4.1 ± 2.9	0.5 ± 0.7	4.1 ± 3.3	0.707
Average number of abortions		0.8 ± 1.2	0	0.4 ± 0.5	1.8 ± 1.3	0.8 ± 1.1	0.311
Number of previous ART attempts		1.1 ± 1.4	0	2.6 ± 2.1	1.0 ± 1.4	1.5 ± 1.7	**0.036** ^c^
Cause of infertility	Maternal	10 (19.6%)	0	4 (40%)	1 (11.1%)	15 (20.3%)	0.303
	Paternal	6 (11.8%)	1 (50%)	4 (40%)	1 (11.1%)	12 (16.2%)	0.066
	Both	10 (19.6)	1 (50%)	2 (20%)	0	13 (17.6%)	0.815
	Unknown	25 (49%)	0	0	7 (77.8%)	34 (45.9%)	–
Abnormal sperm count		9 (17.6%)	1 (50%)	4 (33.3%)	1 (50%)	15 (20.3%)	0.227
Maternal mean age at ART (years)		36.5 ± 4.6	38	35.4 ± 4.0	34.1 ± 4.5	35.9 ± 4.4	0.479
Mean paternal age at ART (years)		38.6 ± 5.1	45	38.6 ± 4.1	41.3 ± 8.3	39.1 ± 5.4	0.985
Average number of oocytes retrieved		8.4 ± 5.2	4	8.9 ± 3.0	8.0 ± 7.1	8.3 ± 4.7	0.822
Gamete freezing^b^		3/25 (12.0%)	1/2 (50%)	0/9	0/2	4/38 (10.5%)	0.276
Embryo freezing^b^		11/25 (44.0%)	1/2 (50%)	2/9 (22.2%)	1/2 (50%)	15/38 (39.5%)	0.249
Average number of embryos obtained		4.5 ± 4.2	2	4.8 ± 2.6	3.5 ± 3.5	4.4 ± 3.7	0.866
Technique used	Stimulation only	1 (2%)	0	0	0	1 (1.4%)	1
	IUI	1 (2%)	0	0	0	1 (1.4%)	1
	IVF	12 (23.5%)	1 (50%)	6 (50%)	1 (25%)	20 (27.0%)	0.068
	ICSI	23 (45%)	1 (50%)	5 (41.7%)	3 (75%)	32 (43.2%)	0.830
	Not available	14 (27.5%)	0	1 (8.3%)	0	20 (27%)	–
Gamete origin	Homologous	23 (45.1%)	1 (50%)	8 (66.7%)	2 (22.2%)	34 (45.9%)	0.178
	Heterologous	4 (7.8%)	1 (50%)	1 (8.3%)	1 (11.1%)	7 (9.5%)	
	Not available	24 (47.1%)	0	3 (25%)	6 (66.7%)	33 (44.6%)	–
Number of embryos transferred		1.7 ± 0.7	1.5 ± 0.7	2.1 ± 0.6	1.0 ± 0.0	1.8 ± 0.7	0.127
Twin pregnancy	Twin at conception	15 (29.4%)	1 (50%)	1 (8.3%)	2 (22.2%)	19 (25.7%)	0.131
	Twin at birth	10 (19.6%)	1 (50%)	0	0	11 (14.9%)	0.186
	Monozygote	2/15 (13.3%)	1/1 (100%)	0/1	0/2	3/19 (15.8%)	0.696
	Dizygote	13/15 (86.7%)	0/1	1/1 (100%)	2/2 (100%)	16/19 (84.2%)	
Pregnancy complications		19 (37.6%)	1 (50%)	3 (25%)	2 (22.2%)	25 (33.8%)	0.423
Abnormal prenatal ultrasound		15 (29.4%)	0	4 (33.3%)	0	19 (25.7%)	0.071
Gestational age		36.4 ± 2.6	37.2 ± 1.2	37.0 ± 4.1	35.4 ± 3.1	36.4 ± 2.9	0.546
Weight at birth (SDS)		1.3 ± 1.7	0.0 ± 1.0	1.2 ± 1.7	1.1 ± 2.9	1.2 ± 1.9	0.908
Lenght at birth (SDS)		1.1 ± 1.5	1.0 ± 0.9	0.7 ± 1.3	0.0 ± 1.4	0.8 ± 1.5	0.443
Head circumference at birth (SDS)		0.5 ± 1.5	0.0 ± 1.6	0.0 ± 0.9	0.3 ± 1.8	0.4 ± 1.4	0.391
Birth complications		8 (15.7%)	0	1 (8.3%)	0	9 (12.2%)	0.513

Abbreviations: IC1‐GoM, imprinting center 1 gain of methylation; IC2‐LoM, imprinting center 2 loss of methylation; SDS, standard deviation score; UPD(11)pat, chromosome 11 paternal uniparental disomy.

^a^ The *p*‐value refers to the comparison between the subgroups with IC2‐LoM and UPD(11)pat.

^b^ Data available only in 38 patients.

^c^ Statistically significant.

Table 3 summarizes the studies retrieved from literature providing genotype and phenotype data of ART‐BWSp patients (*n* = 168).^10^, ^20^, ^21^, ^22^, ^23^, ^27^, ^28^, ^29^, ^30^, ^31^, ^32^, ^33^, ^34^, ^35^, ^36^, ^37^

**TABLE 3:** Studies in the literature analyzing the association between the Beckwith‐Wiedemann spectrum (BWSp) and assisted reproductive techniques (ART)

	BWSp ART/non‐ART	ART‐BWSp cases with molecular testing	Molecular defects found in ART‐BWSp	Kind of ART used
DeBaun et al., 2003^21^	7/0	6	4 IC2‐LoM, 1 IC2‐LoM + IC1‐GoM^a^, 1 negative	IVF, ICSI
Maher et al., 2003^20^	6/149	2	2 IC2‐LoM	IVF, ICSI
Gicquel et al., 2003^22^	6/149	6	6 IC2‐LoM	IVF, ICSI
Halliday et al., 2004^10^	4/37	3	3 IC2‐LoM	IVF, ICSI
Rossignol et al., 2006^33^	11/40	11	11 IC2‐LoM	IVF, ICSI
Sutcliffe et al., 2006^34^	11/79	8	8 IC2‐LoM	ICSI, IVF, ovulation induction
Bowdin et al., 2007^35^	–	1	1 IC2‐LoM	IVF, ICSI
Doornbos et al., 2007^36^	6/71	4	4 IC2‐LoM	IVF, ICSI
Lim et al., 2009^37^	25/112	25	24 IC2‐LoM, 1 negative	IVF, ICSI
Hiura et al., 2012^27^	6/70	1	1 IC2‐LoM	ICSI
Tee et al., 2013^28^	14/187	14	14 IC2‐LoM	–
Tenorio et al., 2016^41^	17/156	17	15 IC2‐LoM, 2 negative	IVF, ICSI
Johnson et al., 2018^29^	16/40	16	15 IC2‐LoM, 1 UPD(11)pat	IVF
Duffy et al., 2019^23^	40/208	40	34 IC2‐LoM, 3 IC1‐GoM, 3 UPD(11)pat	IUI, IVF, ICSI
Hattori et al., 2019^30^	7/117	5	3 IC2‐LoM, 1 IC1‐GoM, 1 negative	Ovulation induction, IVF, ICSI
Hara‐Isono et al., 2020^31^	8/31	8	6 IC2‐LoM and 2 IC1‐GoM	IVF, ICSI, FER
Eltan et al., 2020^32^	1/0	1	1 IC2‐LoM	IVF
Total	186/1446	168	152 IC2‐LoM, 5 UPD(11)pat, 6 IC1‐GoM, 5 negative	–

Abbreviations: ART, artificial reproduction techniques; BWSp, Beckwith‐Wiedemann spectrum; IC1‐GoM, imprinting center 1 gain of methylation; IC2‐LoM, imprinting center 2 loss of methylation; ICSI, intracytoplasmatic sperm injection; IUI, intrauterine insemination; IVF, in‐vitro fertilization; UPD(11)pat, chromosome 11 paternal uniparental disomy.

^a^ UPD(11)pat was excluded.

**FIGURE 1:**
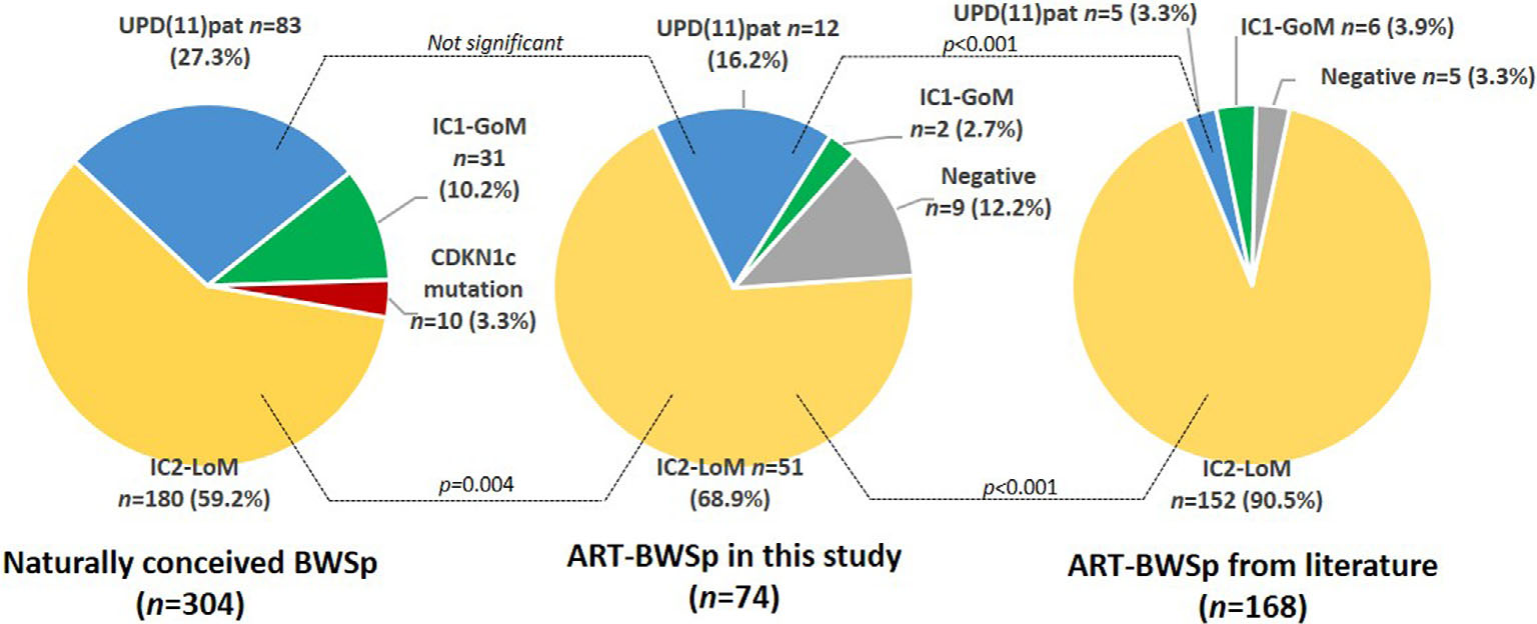
Comparison of the different molecular subtypes of the BWSp in the cohorts of patients with BWSp naturally conceived (left panel), conceived through artificial reproductive technology (ART) in our study (central panel) and from literature (right panel). BWSp, Beckwith–Wiedemann spectrum [Colour figure can be viewed at wileyonlinelibrary.com]

Figure 1 reports the distribution of the molecular subgroups in our non‐ART‐BWSp and ART‐BWSp cohorts, and in the literature‐derived ART‐BWSp cohort, including only patients with positive molecular test. IC2‐LoM cases were 78.5% versus 93.2% in the literature‐derived ART‐BWSp (*p* < 0.001) and 59.2% in our non‐ART‐BWSp cohort, respectively. The distribution of the molecular subtypes in our ART‐BWSp cohort was significantly different from that of the literature‐derived ART‐BWSp cohort. This difference with previous literature is due to a higher fraction of UPD(11)pat cases in our ART‐BWSp cohort compared to the literature (18.5% vs. 3.4%, *p* < 0.001). Overall, both the distribution of the molecular subgroups in the ART‐BWSp cohort was different from that of the non‐ART cohort (*p* = 0.018), and this was mostly due to a higher fraction of IC2‐LoM cases (*p* = 0.004); conversely, the UPD(11)pat frequency in our ART‐BWSp cohort (18.5%) was not significantly different from that observed in the non‐ART BWSp cohort (27.3%), but it significantly higher than that reported in the literature ART‐BWSp one (3.0%, *p* < 0.001). IC1‐GoM was underrepresented in both the ART cohorts compared to the naturally conceived BWSp cohort (8/230, 3.5% vs. 31/304, 10.2%, *p* = 0.004). No patient with *CDKN1C* mutation was observed in the ART cohorts.

Table 4 reports the comparison of the clinical features of cases with IC2‐LoM and UPD(11)pat in the ART‐BWSp and naturally conceived BWSp patients. We observed that omphalocele was less frequent in the IC2‐LoM ART‐BWSp subgroup than that in the naturally conceived patients with the same molecular defect (13.7% vs. 28.9%, *p* = 0.028); however, minor abdominal wall defects were more common in the former than in the latter group (52.9% vs. 36.7%, *p* = 0.036). Moreover, neonatal overgrowth was less common and birth parameters were lower in the ART‐BWSp patients than in those conceived naturally (33.3% vs. 63.9%, *p* = 0.044). Also the auricular anomalies were less represented in the ART‐IC2‐LoM group than in the non‐ART‐IC2‐LoM one (31.4% vs. 50.0%, *p* = 0.018). Overall, twin births were more common in the ART‐BWSp than in the naturally conceived BWSp patients (14.9% vs. 3.3%, *p* < 0.001); among the twins, there was a significant difference between the IC2‐LoM patients conceived after ART (19.6%) and those conceived naturally (5.6%, *p* = 0.002). Finally, gestational age was lower among the ART‐BWSp patients than those conceived naturally (36.4 ± 2.9 vs. 37.2 ± 2.5 weeks, *p* = 0.017).

**TABLE 4:** Comparison between patients with Beckwith‐Wiedemann spectrum (BWSp) born from artificial reproduction techniques (ART) (our study cohort) and patients born from natural conception (historical cohort^16^)

	IC2‐LoM ART	IC2‐LoM not‐ART	*p*	UPD(11)pat ART	UPD(11)pat non‐ART	*p*	Total ART	Total not‐ART	*p*
*n*	51	180	**–**	12	83	–	74	304	–
Neonatal hypoglycemia/hyperinsulinism	20 (39.2%)	55 (30.6%)	0.244	6 (50.0%)	29 (34.9%)	0.312	34 (46.0%)	96 (31.6%)	**0.020** ^a^
Macroglossia	42 (82.5%)	158 (87.8%)	0.316	7 (58.3%)	56 (67.5%)	0.531	56 (75.7%)	249 (81.9%)	0.223
Abdominal wall defects	34 (66.7%)	118 (65.6%)	0.883	7 (58.3%)	41 (49.4%)	0.563	49 (66.2%)	188 (61.8%)	0.485
*Omphalocele*	7 (13.7%)	52 (28.9%)	**0.028** ^a^	2 (16.7%)	6 (7.2%)	0.271	9 (12.2%)	68 (22.4%)	**0.051** ^a^
*Umbilical hernia/diastasis recti*	27 (52.9%)	66 (36.7%)	**0.036** ^a^	5 (41.7%)	35 (42.2%)	0.974	40 (54.1%)	119 (39.1%)	**0.020** ^a^
Lateralized overgrowth	26 (51.0%)	85 (47.2%)	0.635	11 (91.7%)	69 (83.1%)	0.449	42 (56.8%)	168 (55.3%)	0.817
Organ enlargement	9 (17.6%)	49 (27.2%)	0.164	3 (25.0%)	30 (36.1%)	0.449	14 (18.9%)	101 (33.2%)	0.016
Ear pits or creases	16 (31.4%)	90 (50.0%)	**0.018** ^a^	7 (58.3%)	33 (39.8%)	0.223	30 (40.5%)	136 (44.7%)	0.514
Angioma at the glabella	27 (52.9%)	87 (48.3%)	0.561	4 (33.3%)	28 (33.7%)	0.978	37 (50.0%)	128 (42.1%)	0.219
Polyhydramnios	8 (15.7%)	26 (14.4%)	0.825	2 (16.7%)	11 (13.3%)	0.748	10 (13.5%)	48 (15.8%)	0.626
Neonatal macrosomia	22 (43.1%)	102 (56.7%)	0.087	4 (33.3%)	53 (63.9%)	**0.044** ^a^	28 (37.8%)	189 (62.2%)	<0.001*
Malignant tumors	0 (0%)	3 (1.7%)	0.998	2 (16.7%)	13 (15.7%)	0.929	2 (2.7%)	23 (7.6%)	0.131
Twin delivery	10 (19.6%)	10 (5.6%)	**0.002** ^a^	0	0	–	11 (14.9%)	10 (3.3%)	**<0.001** ^a^
Gestational age	36.4 ± 2.6	37.1 ± 2.5	0.082	37.0 ± 4.1	38.1 ± 1.7	0.290	36.4 ± 2.9	37.2 ± 2.5	**0.017** ^a^
Weight at birth (SDS)	1.2 ± 1.6	1.8 ± 1.5	**0.049** ^a^	1.2 ± 1.7	2 ± 1.5	0.093	1.2 ± 1.9	2.1 ± 1.8	**<0.001** ^a^
Lenght at birth (SDS)	1.1 ± 1.5	1.5 ± 1.6	0.112	0.7 ± 1.3	1.4 ± 1.4	0.106	0.8 ± 1.5	1.6 ± 1.6	**<0.001** ^a^
Head circumference at birth (SDS)	0.5 ± 1.5	1.0 ± 1.3	**0.020** ^a^	0.0 ± 0.9	0.8 ± 1.1	0.018	0.4 ± 1.4	1 ± 1.3	**<0.001** ^a^

Abbreviations: IC1‐GoM, imprinting center 1 gain of methylation; IC2‐LoM, imprinting center 2 loss of methylation; SDS, standard deviation score; UPD(11)pat, chromosome 11 paternal uniparental disomy.

^a^ Statistically significant.

**FIGURE 2:**
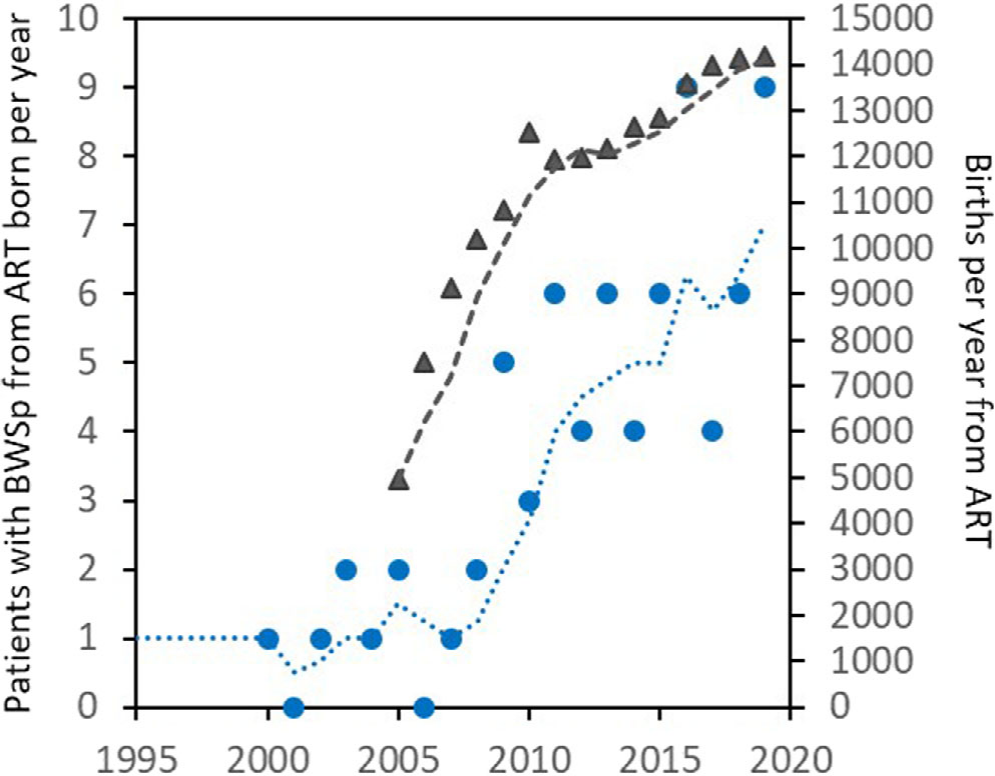
Trend over the last 25 years of the total births per year of children conceived through artificial reproductive technologies (ART) from the Italian ART Registry (years 2005–2019, gray triangles) and ART patients with Beckwith‐Wiedemann spectrum (BWSp, blue circles). Dotted lines represent respective mobile averages: ART‐BWSp births per year increased over time since 2005 (*r* ^2^ = 0.657, *p* < 0.001) [Colour figure can be viewed at wileyonlinelibrary.com]

Since its institution in 2005 to the last registry data release in 2019, 172 568 children from ART were registered in the Italian ART Registry, including the 67 patients with BWSp of our cohort. This allowed to calculate a minimum prevalence of BWSp of 1 in 2575 live births. Over this period, the number of patients with BWSp conceived though ART born each year showed a constant increase over the years (*r* ^2^ = 0.657, *p* < 0.001), paralleling that of births after ART (Figure 2). The comparison between our ART‐BWSp cohort and the children conceived after ART in the 2005 to 2018 time‐period showed that the patients with ART‐BWSp were more commonly premature (42.5%) than both those from the ART Italian Registry (20.8%, *p* = 0.022) and the naturally conceived BWSp children (28.6%, *p* < 0.001). The rate of twin births in our ART‐BWSp cohort (14.9%) and in the Italian ART Registry (16.7%) was similar, but both were higher than those observed in the naturally conceived BWSp patients (3.3%, *p* = 0.524).

## 4 DISCUSSION

Since its first report, many data on the association between ART and BWSp have accumulated: most studies concluded that the ART rate in the BWSp cohorts is higher than in the general population indicating that the risk of BWSp in the children born after ART is 10‐folds than those conceived naturally.^6^ To gain further insights into the relationship between ART and BWSp, here we report the molecular and clinical features of a large cohort of patients with ART‐BWSp and compare this cohort with our historical cohort of non‐ART‐BWSp, with an ART‐BWSp derived from published data, and with the non‐BWSp‐ART children of the Italian ART Registry.

These results contrast with previous reports stating that the molecular abnormality of ART‐BWSp patients is almost exclusively (>90%) IC2‐LoM.^37^ Instead, in our ART‐BWSp cohort the fraction of cases with IC2‐LoM was 78.5%, much closer to that of the naturally conceived patients in our (59%) and other cohorts (50%–60%).^19^ The differences in the molecular breakdown between our ART‐BWSp cohort and the cohort of the literature largely result from a higher prevalence of UPD(11)pat: this molecular defect is nearly 16% in our ART‐BWSp group, but only 3% previously reported ART‐BWSp cases. This discrepancy could be due to a selection bias (i.e., previous studies mostly focused on methylation anomalies) or lower sensitivity of diagnostic tests in the older studies. For instance, a possible explanation could be the age at patients' evaluation: earlier studies and cohorts from laboratory referrals might have likely investigated younger patients (perhaps after birth) and not based on a lasting follow‐up: this might have led to underdiagnose cases presenting later in childhood, as typically happens in patients with mild UPD(11)pat. Another possibility is that mosaic UPD(11)pat might have been incorrectly diagnosed as IC2‐LoM in the older studies. Copy‐neutral segmentally restricted and mosaic UPD is thought to arise post‐zygotically from homologous recombination due to repair of double‐stranded DNA breaks. This type of UPD is quite rare in congenital diseases and in BWS is associated with cell growth advantage due to duplication of the paternal and loss of the maternal imprinted 11p15.5 genes. It is possible that the characteristics of the parents undergoing ART (e.g., health conditions, cause of subfertility) may predispose to this mitotic error in the embryo.

Many of the genotype–phenotype correlations previously reported in the BWSp^13^, ^15^, ^17^, ^23^, ^38^ were grossly confirmed in our ART‐BWSp cohort as well. In particular, lateralized overgrowth and tumors were more common in the subgroup with UPD(11)pat, renal/ureteral anomalies less common in the IC2‐LoM group, as observed previously.^39^, ^40^ However, some features were less common and less severe in the ART patients' group than in the naturally conceived patients. Patients with ART‐BWSp tended to have less severe abdominal defects with a lower incidence of omphalocele, less commonly had macrosomia, and showed lower birth parameters, and fewer cases had ear signs. A milder phenotype in cases from ART with respect to the naturally conceived ones was consistent with previous observations.^41^ These differences were mostly evident in the IC2‐LoM group, although a tendency was also observed in the UPD(11)pat one. Although the milder phenotype observed in the ART‐BWSp group could simply result from the smaller sample size, on the other hand, the lower incidence of major abdominal wall defect could be the result of probably higher rate of pregnancy termination in cases with severe malformation diagnosed at the prenatal ultrasound. The smaller fetal size (and the lower rate of overgrowth) could be attributable to an average more diseased pregnancy in ART, to an average higher parental age, or to the higher incidence of multiple pregnancies in this group. Accordingly, the ART group also had a lower mean gestational age at birth with a higher incidence of preterm births. The milder phenotype we observed in the IC2‐LoM born from ART and naturally conceived, however, could also result from a different timing of onset of the methylation defect during the blastogenesis, resulting in a less represented mosaic in patients with ART‐BWSp. However, we did not observe any correlation between the kind of technique used, nor with other variables as cause of infertility or parental age.

A higher rate of twin births was observed in our cohort, compared with that of naturally conceived patients with BWSp (14.9% vs. 3.3%) and no twins births were observed in the subgroup with UPD(11)pat. These observations further corroborate the hypothesis of the close interconnection between methylation abnormalities, maternal infertility, oocyte abnormalities, disruption of early embryo developmental stages, and twinning.^42^, ^43^

Finally, this study shows that the incidence of patients with BWSp conceived throught ART increase over time paralleling the trend of ART in Italy over the last decades and may therefore further change in the future consistent with ART usage. Although the ART‐BWSp cohort we collected in this study is far from including all the Italian BWSp patients conceived throught ART, we used our data to estimate a minimum prevalence of BWSp in ART‐conceived children, resulting in nearly 1:2500 live births. This estimate is less precise than that we previously calculated on the basis of regional data based on merged ART/BWSp patients' registries (1:1126),^6^ but provides the first minimum prevalence appraisal on a national basis.

In conclusion, this study describes the clinical and molecular features of the largest cohort of patients with BWSp born though ART, making a comparison with previous literature, with naturally conceived patients with BWSp, and with the general population from ART. These results allow spotting some new insights into the connection between ART and BWSp. First, the breakdown of the various molecular subtypes of BWSp is not greatly different in the ART and the naturally conceived patients as previously thought, while UPD(11)pat fraction is similar in the two groups, in contrast with previous reports. Second, there is evidence that patients with BWSp born though ART might have a milder phenotype. Finally, our data first show a progressive increase in the prevalence of BWSp over time, paralleling that of ART usage in the last decades.

## CONFLICT OF INTEREST

The authors declare no conflict of interest.

### PEER REVIEW

The peer review history for this article is available at https://publons.com/publon/10.1111/cge.14193.

## Data Availability

The data that support the findings of this study are available from the corresponding author upon reasonable request.
